# Induction of Humoral and Cellular Immunity After SARS‐CoV‐2 JN.1 Vaccination in Individuals With and Without Prior Infection

**DOI:** 10.1002/eji.70232

**Published:** 2026-06-23

**Authors:** Caroline Diener, Rebecca Urschel, Saskia Bronder, Candida Guckelmus, Johannes Eckel, Franziska Hielscher, Denisa Bojkova, Sandra Ciesek, Marek Widera, Tina Schmidt, Martina Sester

**Affiliations:** ^1^ Department of Transplant and Infection Immunology, PharmaScienceHub Saarland University Homburg Germany; ^2^ Institute For Medical Virology Goethe University Frankfurt University Hospital Frankfurt Frankfurt am Main Germany; ^3^ German Center for Infection Research (DZIF) Partner Site Frankfurt Frankfurt am Main Germany; ^4^ Fraunhofer Institute for Molecular Biology and Applied Ecology Branch Translational Medicine and Pharmacology Frankfurt am Main Germany; ^5^ Center For Gender‐specific Biology and Medicine (CGBM) Saarland University Homburg Germany

**Keywords:** CD4 T cell, CD8 T cell, cross‐reactivity, JN.1 vaccination, omicron JN.1, SARS‐CoV‐2

## Abstract

The continuous evolution of SARS‐CoV‐2 raises concerns about immune escape from preexisting immunity. The monovalent JN.1adapted mRNA vaccine was developed to better match circulating variants, yet data on its ability to induce and broaden humoral and cellular immunity in individuals with or without prior infection remain limited. We recruited 37 immunocompetent adults before and two weeks after JN.1 vaccination to assess vaccine‐induced immunity. Spike‐specific CD4 and CD8 T cells were quantified following stimulation with spike‐derived peptides from the parental strain, XBB.1.5, and JN.1, and their CTLA‐4 expression and cytokine profiles were analyzed by flow cytometry. Spike‐specific IgG and neutralizing activity against authentic parental, XBB.1.5, JN.1, and KP.3.1.1 isolates were also measured. JN.1 vaccination significantly increased spike‐specific CD4^+^ and CD8^+^ T‐cell frequencies with comparable cytokine profiles across variants and enhanced CTLA‐4 expression. IgG levels and neutralizing titers rose markedly, with the strongest relative increases against Omicron lineage variants. Prior infection was associated with higher neutralizing titers but did not influence T‐cell responses. Influenza vaccine co‐administration had no adverse effect on JN.1 immunogenicity. These findings indicate that hybrid immunity enhances antibody‐mediated protection, while robust, cross‐reactive T‐cell responses may contribute to sustained protection against severe disease irrespective of infection history.

## Graphical abstract

JN.1 vaccination induced strong, cross‐reactive CD4 and CD8 T‐cell responses and markedly boosted neutralizing antibodies, especially against omicron variants. Influenza vaccine coadministration did not impair immunogenicity. Prior SARS‐CoV‐2 infection was associated with higher baseline and postvaccine neutralizing activity, while cellular responses remained unaffected by infection history.

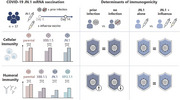

**Abbreviations**
COVID‐19: coronavirus disease 19
CTLA‐4: cytotoxic T lymphocyte‐associated protein 4
DMSO: dimethyl sulfoxide
ELISA: enzyme‐linked immunosorbent assay
IFNγ: interferon gamma
IL: interleukin
MHC: major histocompatibility complex
mRNA: messenger ribonucleic acid
SARS‐CoV‐2: severe acute respiratory syndrome coronavirus 2
SEB: *Staphylococcus aureus* enterotoxin B
TNF: tumor necrosis factor

## 1 Introduction

COVID‐19 vaccinations made an indispensable contribution to containing the SARS‐CoV‐2 pandemic and played a key role in reducing the risk of COVID‐19 infection and disease [1]. A high mutagenic potential and the ongoing emergence of new virus variants still require regular adaptation of existing vaccines to ensure sustained protection, especially of high‐risk patients such as health care workers, the elderly, or immunocompromised persons [2]. In these risk groups, COVID‐19 vaccinations are now recommended as seasonal vaccinations, which are often co‐administered with seasonal influenza vaccines.

The omicron variant, which first appeared in 2021 and as of now comprises five lineages (BA.1‐BA.5), acquired distinct characteristics associated with higher transmissibility, superior infectivity, and improved immune evasion as compared with the parental SARS‐CoV‐2 strain [3, 4]. Later, the XBB‐sublineages, including XBB.1.5, evolved from omicron BA.2 sublineages and rapidly became the major cause of infections worldwide [5]. This was followed by the JN.1, which harbors an accumulation of more than 30 spike protein‐related mutations and thus significantly differs from previous variants [6]. As a consequence, vaccine recommendations for the 2024/2025 season were targeting the JN.1 lineage [2].

The majority of the human population has meanwhile been multiply exposed to SARS‐CoV‐2 antigens by natural infections and/or vaccinations. A major concern with repeated immunizations is that immune imprinting by previous vaccinations may bias future immune responses against SARS‐CoV‐2 [7]. Since initial immunization events were generally induced by vaccines covering the parental spike, first observations on the variant‐adapted vaccines did show a booster effect on antibody levels but reduced neutralizing capacity against common omicron variants [8, 9, 10]. Evidence from subsequent analyses suggests that repeated omicron contacts could reshape neutralizing antibody repertoires, thereby overriding immune imprinting by the ancestral strain [11]. Nevertheless, it remains unclear whether this applies to both antibody and T‐cell immunity, and whether new JN.1 variant‐specific booster vaccines permit sufficient adaptation of the immune responses to new lineages [7, 8, 9, 10].

So far, most studies evaluating the immunogenicity of the JN.1 booster vaccines have focused primarily on humoral immune responses [12, 13, 14], and have not stratified between individuals with and without prior infection. We therefore performed a detailed characterization of spike‐specific humoral and cellular immunity before and after JN.1 mRNA vaccination. To account for ongoing viral evolution, reactivity against the parental strain was compared with that against current omicron lineages. All data were tested against confounders such as a history of previous infection or influenza vaccine co‐administration.

## 2 Results

### 2.1 Study Group, Experimental Setup, and Differential Blood Counts

Thirty‐seven immunocompetent individuals were enrolled (13 males and 24 females, mean age 48±13 years). Of these, 26 reported a known history of SARS‐CoV‐2 infection (confirmed by quantitative PCR or rapid antigen testing and/or a positive nucleocapsid protein (NCP) IgG serology). Twenty‐one individuals had received a homologous series of mRNA vaccinations in the past, while 16 had a history of a heterologous vector/mRNA vaccination. The total number of previous SARS‐CoV‐2 immunization events, including both infections and vaccinations, ranged from three to seven. Among the 37 JN.1 vaccinated subjects, 25 (67.6%) had received simultaneous influenza vaccination. Basic demographic information on the study group is summarized in Table 1.

**TABLE 1:** Overview of demographic characteristics and blood cell counts of the study population.

Parameter	JN.1 vaccination cohort *n* = 37		
Years of age (mean ± SD)	48.1 ± 13.4		
Sex^a^, *n* (%)			
Male	13 (35.1%)		
Female	24 (64.9%)		
Previous SARS‐CoV‐2 vaccine regimen, *n* (%)			
mRNA homologous	21 (56.8%)		
Vector/mRNA combination	16 (43.2%)		
Persons with known history of COVID‐19 infection, *n* (%)			
Yes	26 (70.3%)		
No	11 (29.7%)		
Nucleocapsid‐IgG positive *n* (%)			
History of COVID‐19 infection	10/26 (27.8%)		
No history of COVID‐19 infection	0/11 (0%)		
Number of previous immunization events^b^			
3	1		
4	6		
5	16		
6	13		
7	1		
Time since last known immunization event, median days (IQR)	356 (164.5)		
Persons with influenza co‐vaccination, *n* (%)			
Yes	25 (67.6%)		
No	12 (32.4%)		
Differential blood cell counts, median cell number (IQR)	Prevaccination (*n* = 33)	Postvaccination (*n* = 36)	*p*‐value
Leukocytes	6800 (2100)	6750 (2225)	0.594
Granulocytes	4046 (1826)	3883 (1702)	0.733
Monocytes	518 (171)	513 (289)	0.139
Lymphocytes	2041 (775)	2096 (806)	0.454

^a^ Self‐declared.

^b^ Including vaccinations or infections; differential blood counts were determined for all individuals pre and post vaccination, except one participant who only had the pre‐vaccination measurement, and four participants who only had the postvaccination blood cell counts determined.

**FIGURE 1:**
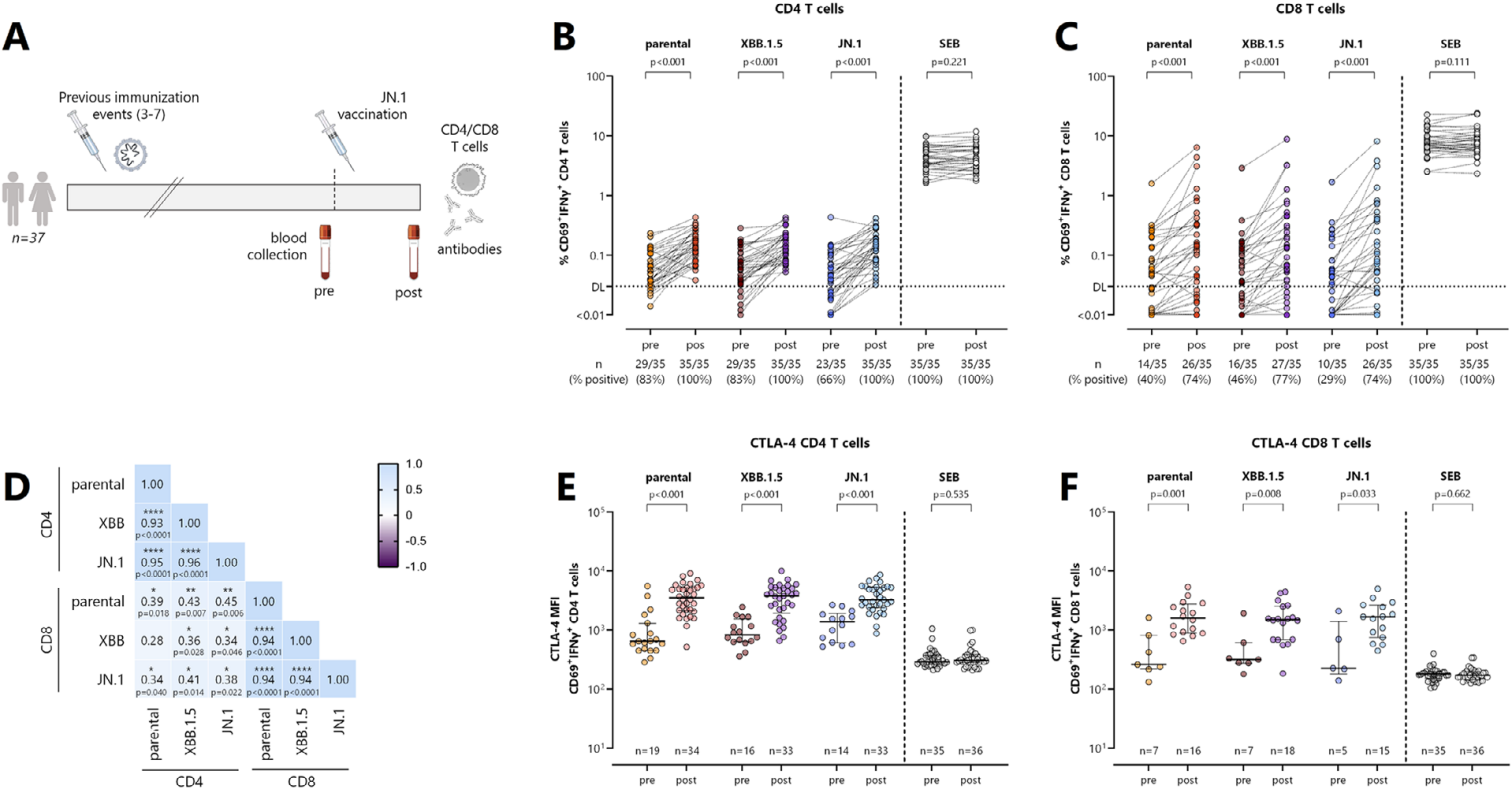
Quantification and characterization of spike‐specific T cells before and after JN.1 booster vaccination. (A) Experimental setup of the study, which included 37 immunocompetent persons with a history of three to seven SARS‐CoV‐2‐related immunization events (including vaccinations and/or infections). Whole blood samples were collected at consecutive time‐points before and two weeks after booster vaccination with a JN.1‐specific mRNA vaccine (Comirnaty JN.1). Humoral and cellular immune responses were assessed, including the identification and phenotypical characterization of antigen‐specific CD4 and CD8 T cells, the quantitation of IgG antibodies, and the determination of their neutralizing activity. Vaccine‐induced humoral and cellular immunity was analyzed before and after vaccination for all individuals except for one person, for whom T‐cell analysis before vaccination was missing. One more person had missing data on JN.1‐specific T cells. The percentages of (B) CD4 and (C) CD8 T cells after stimulation with spike‐derived peptides from the parental strain, XBB.1.5 and JN.1 were determined using flow‐cytometry. Reactive cells were identified based on their co‐expression of CD69 and IFN‐γ, and corrected for background reactivities of the negative control. Analyses of SEB‐reactive cells served as positive controls and spike‐nonspecific T cells throughout the experiments. Detection limits (DL) are indicated by the dotted lines (0.03% for CD4 T cells and 0.06% for CD8 T cells). The numbers (and percentages) of positive samples that were above the DL are indicated. Results before and after monovalent JN.1 vaccination are shown for 35 individuals with paired results. Paired statistical analyses were performed using the Wilcoxon signed‐rank test. *p*‐values <0.05 were considered statistically significant. (D) Correlations between spike‐specific CD4 and CD8 T‐cell levels toward the parental strain and the two variants were analyzed according to Spearman, with correlation coefficients and *p*‐values indicated. CTLA‐4 expression levels (as median fluorescence intensities, MFI) of spike‐specific (E) CD4 and (F) CD8 T cells were determined. All individuals were tested. To ensure robust statistical assessment, the analyses were restricted to samples with at least 20 spike‐specific CD4 or CD8 T cells, with the number of corresponding samples indicated. Statistical analysis was carried out using Mann–Whitney *U* testing. All results are presented as median and interquartile ranges. Panel A was created using illustrations from NIAID NIH BioArt (https://bioart.niaid.nih.gov/).

Blood samples were drawn before and 15 (interquartile range [IQR] 5) days after vaccination. An overview of the experimental setup is shown in Figure 1A. Major blood cell populations were in the normal reference ranges and did not significantly change in response to vaccination (Table 1).

### 2.2 Induction of Spike‐Specific CD4 and CD8 T Cells After JN.1 Booster Vaccination

To quantify and characterize cellular immune responses after JN.1 vaccination, blood cells were stimulated with overlapping peptide pools covering the viral spike proteins of either the SARS‐CoV‐2 parental strain, the variant XBB.1.5, or the JN.1 strain. SEB‐reactive CD4 and CD8 T cells were analyzed as vaccine‐independent controls. Even before vaccination, levels of spike‐specific CD4 T cells were above the detection limit in the majority of individuals (Figure 1B). Corresponding proportions of individuals with detectable antigen‐specific CD4 T cells were 83% (29/35 donors) for both the parental and the XBB.1.5 variants, and 66% (23/35) for the JN.1 variant. The proportions of individuals with detectable spike‐specific CD8 T cells were somewhat lower, with 40%, 46%, and 29% reactive toward the parental, XBB.1.5, and JN.1 spike variants, respectively (Figure 1C). The JN.1 booster vaccination led to a significant increase in both spike‐specific CD4 and CD8 T‐cell levels, which was observed for all tested SARS‐CoV‐2 variants (*p* < 0.001, Figure 1B,C). Spike‐specific CD4 T‐cell levels after vaccination exceeded the detection limit in all individuals, whereas only 74%–77% of individuals reached the detection limit for spike‐specific CD8 T cells. Specific CD4 T‐cell levels toward the vaccine‐derived spike antigen from JN.1 increased by a median of 2.07‐fold (IQR 1.37) and reached 0.14% (IQR 0.13%), which did not significantly differ from increases observed for CD4 T cells toward the parental spike (1.96‐fold (IQR 0.90)) or the XBB.1.5 variant (1.97‐fold (IQR 1.16); *p* = 0.222), indicating substantial cross‐reactivity between the variants (Table S1). Likewise, median levels of JN.1 spike‐reactive CD8 T cells increased 1.83‐fold (IQR 2.07) and reached 0.10% (IQR 0.48%). As with CD4 T cells, this increase was similar for the parental spike (1.50‐fold (IQR 1.33)) and for XBB.1.5 (1.51‐fold (IQR 1.78); *p* = 0.379, Table S1). These effects were vaccine‐specific as median levels of polyclonal CD4 or CD8 T cells after stimulation with SEB did not show any dynamic changes in response to vaccination (1.02‐fold (IQR 0.28) for CD4 T cells, *p* = 0.221; 1.04‐fold (IQR 0.34) for CD8 T cells, *p* = 0.111, Figures 1B and C, Table S1). Within each subpopulation of CD4 or CD8 T cells, there was a significant correlation between the levels of cells specific for the three SARS‐CoV‐2 strains, whereas the correlation between respective CD4 and CD8 T‐cell levels was less tight (Figure 1D).

We further characterized the expression of CTLA‐4 as a marker for T cells with recent specific antigen encounter in vivo [15]. As shown in Figure 1E,F, vaccination led to a significant increase in the median expression of CTLA‐4 on spike‐specific CD4 and CD8 T cells, which was similar for all SARS‐CoV‐2 variants (from *p* < 0.033 to *p* < 0.001). In contrast, CTLA‐4 expression levels on SEB‐stimulated CD4 or CD8 T cells were generally lower and did not significantly change following vaccination (*p* = 0.535 and *p* = 0.662). Notably, CTLA‐4 expression levels on spike‐specific CD4 T cells before vaccination were numerically highest for JN.1‐specific cells, and overall higher than for SEB‐reactive CD4 T cells, which may result from some variable extent of in vivo exposure with SARS‐CoV‐2 before vaccination (Figure 1E,F).

### 2.3 Functional Changes in Spike‐Specific CD4 and CD8 T Cells in Response to Vaccination

**FIGURE 2:**
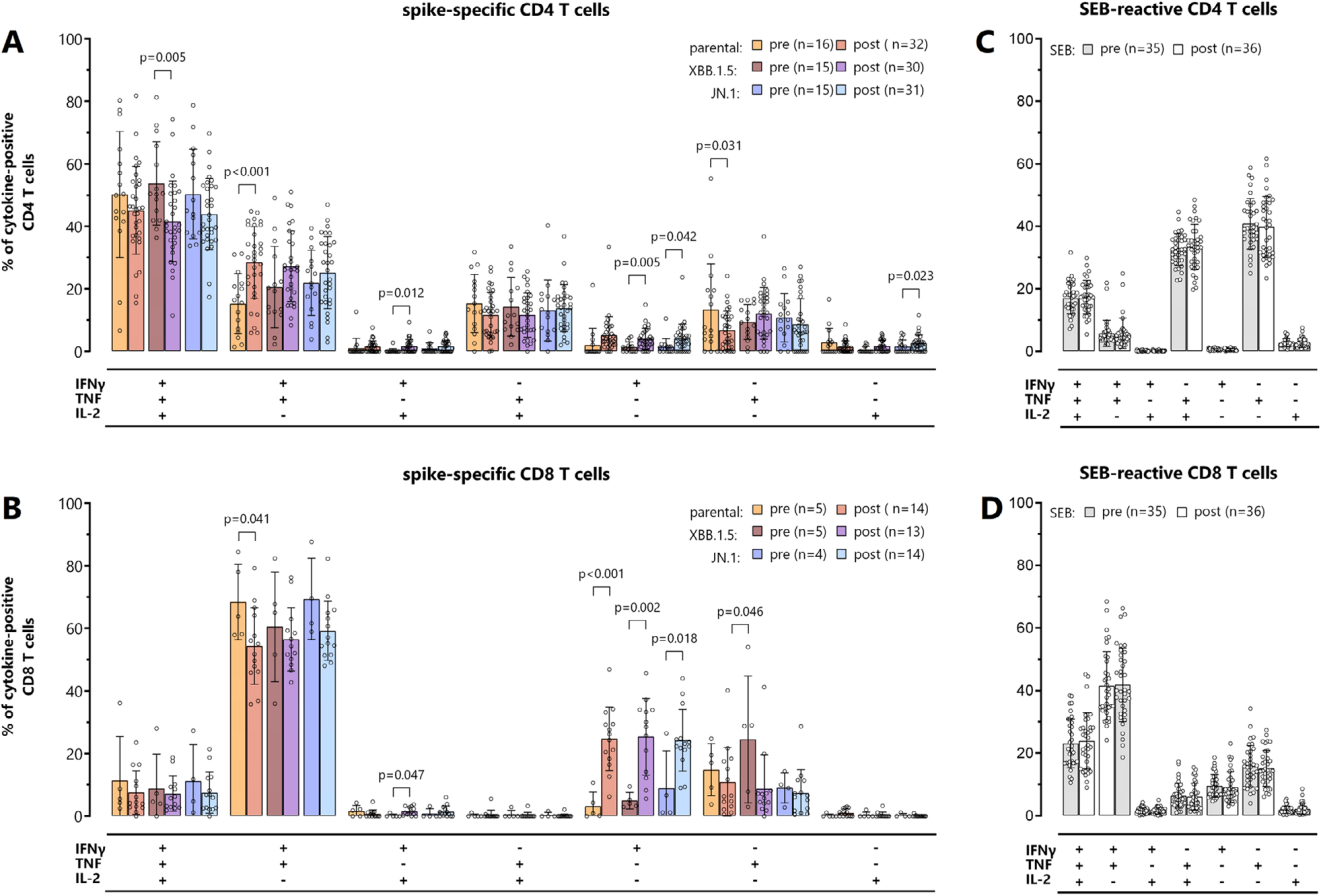
Cytokine expression profiles of spike‐specific and SEB‐reactive T cells before and after JN.1 booster vaccination. Profiles of (A) spike‐specific CD4 and (B) CD8 T cells, and (C) SEB‐reactive CD4 and (D) CD8 T cells were analyzed based on the individual or combined expression of IFN‐γ, TNF, and/or IL‐2 after Boolean gating. All available samples were tested, but to ensure robust statistical assessment, the analyses were restricted to samples with at least 30 cytokine‐expressing cells after background correction with the number of samples indicated. Results are represented as individual values (symbols), including means (bars) with standard deviations (error bars). The unpaired *t*‐test was used to analyze differences in cytokine profiles before and after vaccination.

We further characterized functional features of spike‐specific CD4 and CD8 T cells based on their ability to produce IFN‐γ, TNF, and IL‐2. Boolean gating allowed identification of seven distinct subpopulations producing the cytokines alone or in different combinations (Figure 2). Irrespective of the SARS‐CoV‐2 variant, the majority of spike‐specific CD4 T cells were multifunctional, producing all three cytokines (Figure 2A), whereas the largest fractions of spike‐specific CD8 T cells were double‐positive for IFN‐γ and TNF (Figure 2B). Despite some minor numerical decreases in triple‐positive CD4 T cells with a concomitant increase in IFN‐γ/TNF dual‐positive cells, there were no major changes in the cytokine profiles after JN.1 vaccination. Among CD8 T cells, the major subpopulation of IFN‐γ/TNF dual‐positive cells showed some slight decrease after vaccination, which was associated with a significant increase in IFN‐γ‐single positive T cells. In general, cytokine profiles of CD4 and CD8 T cells were similar across the three variants, but clearly distinct from respective profiles of CD4 or CD8 T cells after polyclonal stimulation with SEB (Figure 2C,D).

### 2.4 Induction of Specific Antibodies and Their Neutralizing Capacities Against Different Variants

**FIGURE 3:**
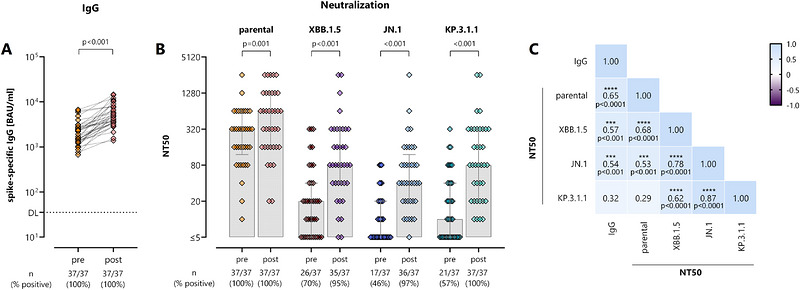
Quantification of spike‐specific IgG and neutralizing antibody levels before and after JN.1 booster vaccination. (A) SARS‐CoV‐2‐specific IgG levels [BAU/mL] toward the parental spike protein and (B) neutralizing antibody activity as expressed by half maximal neutralization titers (NT50, reciprocal dilution factor) toward the parental (FFM7), XBB.1.5, JN.1, and KP.3.1.1 strains were quantified from 37 individuals before and after monovalent JN.1 vaccination. Paired statistical analysis was performed using the Wilcoxon signed‐rank test. Individual results are shown with median and interquartile ranges. The numbers (and percentages) of positive samples with detectable levels are indicated at the bottom of the graph. (C) Correlations between vaccine‐induced IgG levels and neutralizing activity toward the parental strain and the three variants were analyzed according to Spearman, with correlation coefficients and *p*‐values indicated.

We next quantified IgG against the parental spike before and after vaccination, and characterized their neutralizing activity. Overall, levels of IgG against the parental spike showed a significant 2.26‐fold (IQR 1.79) increase in response to booster vaccination from 1701 (IQR 1507) BAU/mL to 6530 (IQR 5334) BAU/mL (*p* < 0.001, Figure 3A; Table S1). Plasma samples were also tested for neutralizing activity against authentic parental SARS‐CoV‐2 and variants XBB.1.5, JN.1, and KP.3.1.1. Despite notable differences between the four strains, JN.1 vaccination led to a significant induction in neutralizing antibody titers (*p* < 0.001). Neutralizing antibodies toward the parental SARS‐CoV‐2 were already detectable prior to vaccination in 100% of individuals. Both before and after vaccination, titers against parental SARS‐CoV‐2 were significantly higher when compared with the three variants (Table S1). The percentage of individuals with neutralizing titers against SARS‐CoV‐2 variants above detection limits rose from 70% to 95% for XBB.1.5, from 46% to 97.0% for JN.1, and from 57% to 100% for KP.3.1.1, respectively. In general, IgG levels and neutralizing antibody titers toward the various SARS‐CoV‐2 strains showed a significant correlation (Figure 3C). An exception was correlations between IgG toward the parental strain and neutralizing antibody titers toward the more distantly related variant KP3.1.1, which did not reach statistical significance (Figure 3C).

### 2.5 Correlation Between Cellular and Humoral Immunity and Confounding Factors

**FIGURE 4:**
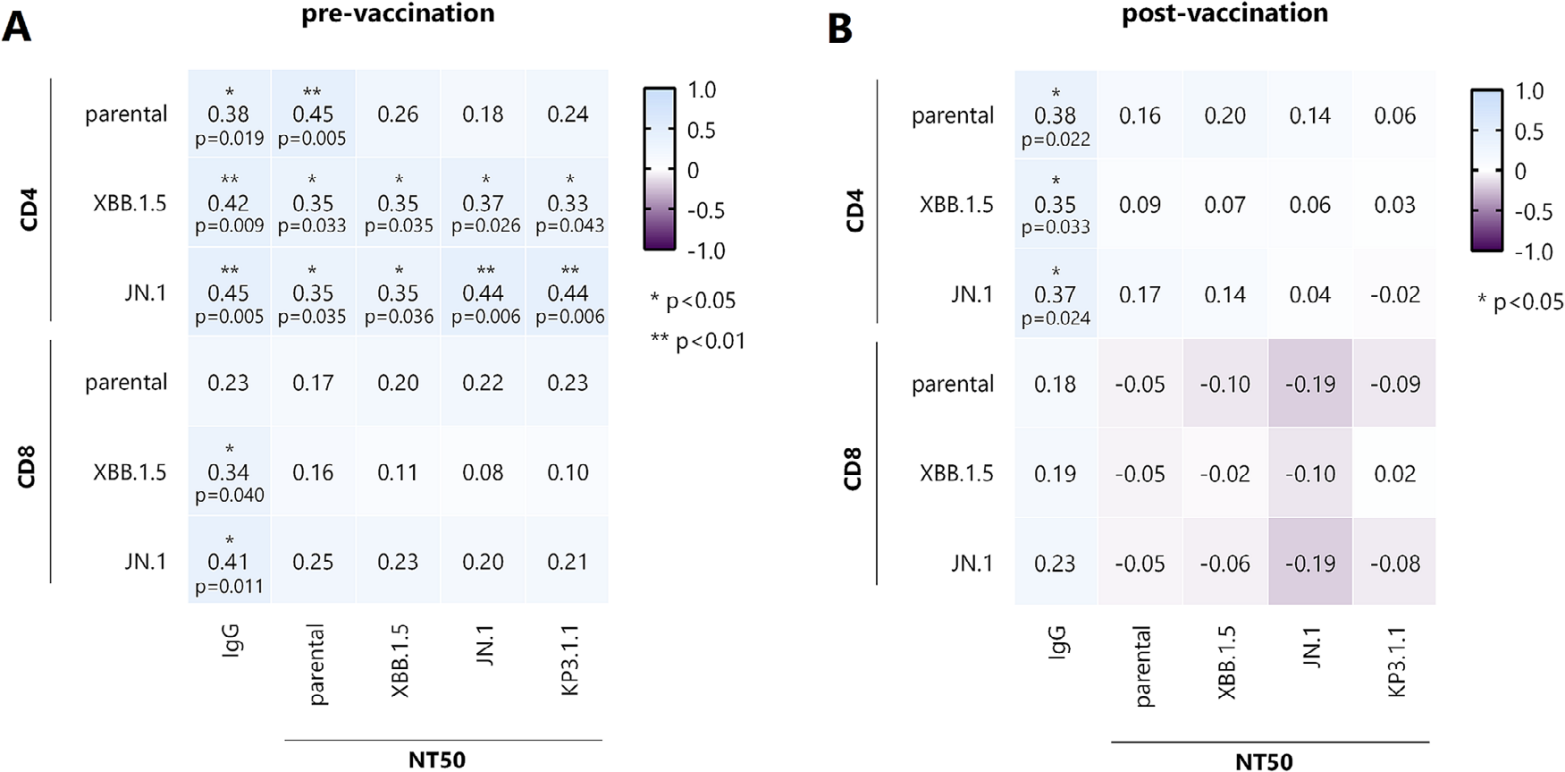
Correlation between spike‐specific T‐cell and antibody levels in response to JN.1 vaccination. Correlation matrix comparing spike‐specific CD4 and CD8 T cells, the parental SARS‐CoV‐2 and variants XBB.1.5., and JN.1, with IgG and neutralizing antibodies toward the parental strain, XBB.1.5, JN.1, and KP3.1.1 among individuals (A) before and (B) after JN.1 vaccination. Correlation coefficients were calculated according to two‐tailed Spearman and displayed using a color code, and *p*‐values represented by stars denoting levels of statistical significance are shown.

We also analyzed correlations between IgG and neutralizing antibody toward the parental strain and the SARS‐CoV‐2 variants with respective spike‐specific CD4 and CD8 T‐cell levels. Both before and after vaccination, IgG levels showed some correlation with spike‐specific CD4 T cells (Figure 4A,B). Moreover, significant correlations before vaccination were found between CD4 T cells and neutralizing antibody titers with the same or similar strain‐specificity (Figure 4A). In contrast, post‐vaccination levels of CD4 T cells did not correlate with neutralizing antibody titers (Figure 4B). Finally, except for some association between IgG levels with XBB.1.5 and JN.1 spike‐specific CD8 T cells before vaccination, there was no correlation between specific CD8 T cells and antibodies, neither pre‐ nor postvaccination (Figure 4A,B).

We finally performed a multivariable linear regression analysis to test as to how vaccine‐induced antibodies and T cells were affected by a history of prior infection (26/37 individuals) or co‐administration of the influenza vaccine (25/37 individuals). As shown in Table 2, age, sex, and influenza vaccination had no effect on any of the vaccine‐induced immune parameters. In contrast, a history of prior SARS‐CoV‐2 infection had a significant effect on neutralizing antibody titers toward all three tested variant strains, but not toward the parental strain (Table 2). Spike‐specific CD4 and CD8 T‐cell levels after vaccination were unaffected by prior infection. Multivariable linear regression of pre‐vaccine levels showed that prior infection also had a confounding effect on IgG titers and on neutralizing antibody activity toward the variant strains (Table S2).

**TABLE 2:** Multivariable regression analyses of vaccine‐induced immune responses.

Dependent variables^a^	Age		Sex			Influenza vaccine co‐administration			History of previous infection		
	Estimate (95% CI)	*p*‐value	Estimate (95% CI)		*p*‐value	Estimate (95% CI)		*p*‐value	Estimate (95% CI)		*p*‐value
			Female [Ref.]	male		No [Ref.]	yes		No [Ref.]	yes	
IgG	−0.006 (−0.013 to 0.001)	0.070	1	−0.045 (−0.231 to 0.141)	0.627	1	0.125 (−0.062 to 0.312)	0.184	1	0.006 (−0.185 to 0.197	0.947
NT50 parental	−0.003 (−0.054 to 0.048)	0.904	1	0.416 (−0.997 to 1.830)	0.553	1	1.067 (−0.357 to 2.490)	0.137	1	0.645 (−0.806 to 2.097)	0.372
NT50 XBB.1.5	−0.012 (−0.068 to 0.043)	0.655	1	0.360 (−1.187 to 1.907)	0.639	1	1.528 (−0.029 to 3.086)	0.054	**1**	**1.710 (0.122 to 3.299)**	**0.036**
NT50 JN.1	−0.036 (−0.077 to 0.006)	0.087	1	0.139 (−1.019 to 1.297)	0.809	1	1.132 (−0.034 to 2.298)	0.057	**1**	**1.934 (0.744** to **3.123)**	**0.002**
NT50 KP.3.1.1	−0.037 (−0.083 to 0.009)	0.112	1	−0.160 (−1.446 to 1.125)	0.801	1	0.933 (−0.362 to 2.228)	0.152	**1**	**2.539 (1.218 to 3.859)**	**<0.001**
CD4 T cells parental	−0.005 (−0.012 to 0.003)	0.240	1	−0.089 (−0.303 to 0.125)	0.402	1	0.039 (−0.177 to 0.255)	0.715	1	−0.177 (−0.397 to 0.043)	0.110
CD4 T cells XBB.1.5	−0.004 (−0.011 to 0.003)	0.307	1	−0.088 (−0.283 to 0.107)	0.365	1	−0.008 (−0.204 to 0.188)	0.935	1	−0.187 (−0.387 to 0.014)	0.067
CD4 T cells JN1	−0.002 (−0.011 to 0.006)	0.562	1	0.009 (−0.242 to 0.260)	0.941	1	−0.084 (−0.339 to 0.170)	0.504	1	−0.114 (−0.359 to 0.131)	0.349
CD8 T cells parental	−0.005 (−0.026 to 0.016)	0.654	1	0.245 (−0.342 to 0.831)	0.402	1	−0.262 (−0.853 to 0.328)	0.373	1	−0.496 (−1.099 to 0.106)	0.103
CD8 T cells XBB	−0.006 (−0.026 to 0.014)	0.558	1	0.274 (−0.296 to 0.844	0.335	1	−0.228 (−0.802 to 0.346)	0.425	1	−0.462 (−1.047 to 0.124)	0.118
CD8 T cells JN.1	−0.003 (−0.025 to 0.019)	0.783	1	0.131 (−0.511 to 0.773)	0.680	1	−0.089 (−0.741 to 0.563)	0.783	1	−0.585 (−1.213 to 0.043)	0.067

*Note*: Shown are *p*‐values of multivariable linear regression analyses with log(10) transformed values for IgG and T cells and log(2) transformed values for NT50 values.

^a^ Parameters refer to spike‐specific IgG [BAU/mL], neutralizing activity (NT50), and spike‐specific CD4 and CD8 T cells [%]; females, no influenza co‐administration, and no prior infection were used as references for categorical parameters; NT50 parental refers to the FFM7 strain. 95% CI, 95% confidence interval.

### 2.6 Vaccination‐Related Adverse Events

Based on a standardized questionnaire, 70% of the individuals reported the occurrence of any type of adverse event within the first seven days after JN.1 vaccination. The most pronounced events were pain at the injection site (57%), fatigue (35%), and headache (27%, Table S3). When stratifying participants according to whether or not they had the influenza vaccine co‐administered, significantly fewer individuals with JN.1/influenza co‐administration reported any adverse events as compared with individuals without (56% vs. 100%, *p* = 0.007; Table S3). Individual local or systemic adverse events did not significantly differ between the subgroups (Table S3).

## 3 Discussion

The SARS‐CoV‐2 subvariant JN.1 and its recent descendants accumulated numerous spike mutations and remain the dominant drivers of worldwide infections [2, 6, 16]. Here, we characterized spike‐specific humoral and cellular immunity following JN.1‐specific mRNA vaccination. We show that the JN.1 vaccine robustly induced both antibodies and T cells. While the vaccine led to a similar induction of cross‐reactive CD4 and CD8 T cells toward JN.1, XBB.1.5, and the parental strain, the relative increase in vaccine‐induced neutralizing antibody activity was more pronounced for all tested omicron variants. Co‐administration of the influenza vaccine did not adversely affect JN.1 vaccine‐induced immune responses. In contrast, a history of COVID‐19 infection was associated with higher levels of both pre‐vaccine and JN.1 vaccine‐induced neutralizing activity.

We show that approximately 50% of tested individuals had pre‐existing antibodies with neutralizing activity toward JN.1, which may in part represent cross‐reactive antibodies induced by previous generations of vaccines [17]; pre‐existing neutralizing activity toward XBB.1.5 was higher and reached response rates of 70%, which may result from the facts that XBB.1.5 was preceding JN.1 and that the majority of our subjects had a history of infection. Moreover, XBB.1.5 constituted the target of the previous vaccines [5, 16, 18], which was also shown to exert some extent of cross‐neutralizing activity toward the JN.1 lineage [19]. In our study, pre‐existing neutralizing antibody activity toward the variants was lowest in individuals without prior infection. In contrast, irrespective of prior infection, the overall highest neutralizing activity was observed toward the parental virus, which suggests that early exposure to parental spike still has a dominant impact on current humoral immunity to SARS‐CoV‐2 [2, 7]. After JN.1 vaccination, pronounced boosting effects on humoral immunity were found for both SARS‐CoV‐2‐specific IgG levels as well as neutralizing activity in the majority of subjects, which is consistent with other recent analyses [12, 13]. The significant increases were not restricted to JN.1 as the original vaccine target but were also found for the parental strain, as well as XBB.1.5 and the JN.1 descendant variant KP.3.1.1. While absolute titers of neutralizing activity toward the parental strain were highest and appeared to reach saturating levels, the relative increase was lower as compared with the tested omicron variants (2‐fold versus 4‐fold). Among the variants, KP.3.1.1 elicited higher response rates and neutralizing antibody titers than JN.1. As KP.3.1.1 shares some amino acid changes with other subsequent JN.1 sublineages, including LP.8.1.1, NB.1.8.1, and XFG [20, 21], this may imply that the JN.1 vaccine might also be cross‐protective against these subvariants.

Limited data exist on JN.1 vaccine‐induced T‐cell immunity in humans. JN.1 reactive CD4 and CD8 T cells were detectable even before JN.1 vaccination in more than 60% and 30% of the tested subjects, respectively. As with vaccines toward the parental strain and variants preceding JN.1, the following characteristics were found for CD4 and CD8 T cells after JN.1 vaccination. Firstly, JN.1‐specific CD4 and CD8 T cells were strongly induced with a higher interindividual variability in spike‐specific CD8 T‐cell levels, which is in line with previous vaccines [22, 23]. Second, despite accumulating mutations, JN.1‐specific CD4 and CD8 T cells showed pronounced cross‐reactivity with the parental strain and the XBB.1.5 variant, which may result from the fact that short peptides presented in MHC class I or II molecules are less affected by mutations than are conformational epitopes of antibodies [2, 24]. In support of cross‐reactivity, respective CD4 and CD8 T‐cell levels are quantitatively similar, which is distinct from variable levels of neutralizing antibodies, and vaccine‐induced T cells toward JN.1 shared similarities in cytokine‐profiles and CTLA‐4 expression with respective T cells specific for the parental spike or XBB.1.5. Similar to our observations, in silico [25] and experimental analyses also found cross‐recognition of the JN.1 precursor BA.2.86 [26, 27] or BA.4/5 [23] and previous strains. Thirdly, unlike antibodies, we show that spike‐specific T‐cell levels are not confounded by prior infection. Interestingly, however, a correlation between spike‐specific CD4 T‐cell levels and neutralizing antibody levels was found before JN.1 vaccination, which was largely driven by individuals with a history of infection. This emphasizes the role of infections in shaping the immune response, and the particular role of CD4 T cells in providing B‐cell help. After JN.1 vaccination, this correlation between CD4 T cells and neutralizing antibodies was no longer observed, which indicates that both CD4 T cells and antibodies undergo a strong expansion to reach more uniform saturating levels [28].

Co‐administration of COVID‐19 and seasonal influenza vaccines is now widely recommended to reduce the burden of severe respiratory infections during the winter months [29, 30]. It remains controversial whether mutual interference between the vaccines may increase adverse events and affect immunogenicity or effectiveness [31]. Although evaluations based on vaccine formulations of the previous years described an increased probability of systemic reactions after co‐administration [32], we did not observe any increased occurrence of adverse events, with even a slightly lower percentage of persons reporting adverse events after co‐administration than after JN.1 vaccination alone. Regarding SARS‐CoV‐2 immunogenicity, some studies described diminished antibody responses upon influenza co‐administration [33], while others did not show any confounding effect on antibody responses [34, 35]. In our study, neither humoral nor cellular immunity was affected by co‐administration.

### 3.1 Data Limitations and Perspectives

Our study has limitations in that the overall sample size, especially of individuals without influenza co‐administration or a history of infection, was low. In addition, immune responses were assessed two weeks after vaccination, which may not capture longer‐term contraction or maturation of immune responses, or information on sustained protection, including the role of absolute or relative increases in vaccine‐induced immunity, which would require larger sample sizes and longer follow‐up. It may also seem increasingly difficult to clearly rule out a history of infection. In this regard, it is reassuring that none of the 11 individuals without a known history of infection had a positive NCP‐IgG result. On the other hand, only 10 out of 26 individuals (38.5%) with a known history of infection had a positive NCP‐serology. While this has been shown to be due to antibody waning over time [36, 37], this emphasizes that estimates of the proportion of individuals with prior infection should not be based on NCP‐antibody testing alone. However, despite suboptimal standards on assigning the infection status, stratification in our study seems reasonable, as levels and patterns of neutralizing antibody activity in individuals with and without prior infection showed distinct differences, which are in line with response patterns observed with previous omicron vaccines [23, 38].

In conclusion, as with previous variant vaccines, the JN.1 vaccine led to a robust induction of spike‐specific CD4 and CD8 T cells and antibodies across multiple SARS‐CoV‐2 variants, which was accompanied by significant increases in neutralizing antibody activity. However, both pre‐ and postvaccination antibody activity were lower in individuals without prior infection. In contrast, cellular immunity was unaffected by prior infection and remained broadly cross‐reactive rather than variant‐specific. This indicates that cellular immunity may provide durable protection from evolving mutants, particularly in preventing severe disease, even when antibody‐mediated protection from infection may be compromised.

## 4 Materials and Methods

### 4.1 Study Participants and Experimental Design

In this observational study, immunocompetent participants were enrolled at Saarland University Medical Center. Heparinized blood samples were collected for the analysis of antigen‐specific T cells and for the preparation of plasma. Differential cell counts were analyzed from EDTA blood. Sampling was conducted before and two weeks after vaccination with the monovalent Comirnaty JN.1 COVID‐19‐mRNA vaccine (Bretovameran, BioNTech SE, Mainz, Germany). All individuals were also offered the quadrivalent influenza vaccine (Influsplit Tetra 2024/2025, GlaxoSmithKline GmbH & Co. KG, München, Germany), which was co‐administered on a voluntary basis. Vaccinations were carried out from August 14, 2024, to November 13, 2024. Histories of previous COVID‐19 vaccinations and infections, age, sex, and vaccine‐related adverse events were self‐reported using a standardized questionnaire. A prior infection was identified either by self‐reported history of SARS‐CoV‐2 infection (confirmed by PCR or rapid antigen test) and/or by a positive nucleocapsid protein (NCP) serology.

### 4.2 Quantification and Characterization of Antigen‐Specific CD4 and CD8 T Cells

Antigen‐specific T‐cell stimulations were conducted from heparinized whole blood as previously described [22, 23, 39]. Samples were stimulated with overlapping peptides covering the SARS‐CoV‐2 spike protein (2 µg/mL, C‐terminal domain with transmembrane domain and N‐terminal receptor binding domain as 15mers with 11aa overlap). Peptide pools from either the parental strain (PM‐WCPV‐S‐1), XBB.1.5 (PM‐SARS2‐SMUT15‐1), or JN.1 (PM‐SARS2‐SMUT22‐1) subvariants (JPT Peptide Technologies GmbH, Berlin, Germany) were dissolved in sterile dimethyl sulfoxide (DMSO) and used from a 1:10 working solution with phosphate‐buffered saline (PBS). A separate reaction was carried out with the addition of the solvent (0.64 % DMSO) to serve as a negative control. Likewise, polyclonal stimulation with 2.5 µg/mL *Staphylococcus aureus* enterotoxin B (SEB, Sigma Aldrich) served as a positive control and as a control population for vaccine nonspecific T cells. Co‐stimulatory antibodies, including anti‐CD28 and anti‐CD49d (1 µg/mL each; BD, Heidelberg, Germany), were added to all stimulatory reactions. Blood samples were incubated for a total of 2 h. Subsequently, 10 µg/mL Brefeldin A was added to inhibit cytokine secretion. After a total of 6 h, 20 mM EDTA was added, followed by treatment with BD lysing solution and a washing step with FACS buffer (PBS‐5%FCS‐0.5%BSA‐0.07%NaN_3_). Fixed cells were permeabilized with 0.2 % saponin in FACS buffer before immunostaining of CD4, CD8, CD69, CTLA‐4 (cytotoxic T‐lymphocyte protein 4), and the cytokines interferon gamma (IFN‐γ), tumor necrosis factor (TNF), and interleukin 2 (IL‐2). Information on antibodies is provided in Table S4. Flow‐cytometric analyses were carried out on a BD FACSCanto II instrument using the BD FACSDiva software v9.0.2 (BD). Co‐expression of CD69 and IFN‐γ was determined for the identification of both antigen‐specific CD4 and CD8 T cells. Quantities of antigen‐specific cells were normalized by subtraction of reactivity in the negative control. Detection limits for antigen‐specific CD4 (0.03%) and CD8 T cells (0.06%) were defined based on the reactivity of the DMSO negative control as described before [23]. Further phenotypical and functional characterization was conducted based on the expression levels of CTLA‐4 and on cytokine‐profiling of IFN‐γ, IL‐2, and TNF, with the gating strategy shown in Figure S1. To ensure robust statistical assessment, evaluation of CTLA‐4 MFI (median fluorescent intensity) was restricted to samples with at least 20 positive events among CD69^+^IFNγ^+^ T cells. Likewise, cytokine analyses were restricted to samples with at least 30 cytokine‐expressing cells after background correction.

### 4.3 Analysis of SARS‐CoV‐2 Specific Antibody Levels and Neutralizing Activity

SARS‐CoV‐2 S‐specific IgG antibody titers were quantified using SARS‐CoV‐2 QuantiVac ELISA (EUROIMMUN Medizinische Labordiagnostika AG, Lübeck, Germany), according to the manufacturer's instructions. Antibody binding units [BAU/ml] below 25.6 were classified as negative. Levels between 25.6 and 35.2 BAU/mL were considered intermediate, and values above 35.2 as positive. SARS‐CoV‐2‐specific IgG toward the nucleocapsid protein (NCP) was identified using the anti‐SARS‐CoV‐2‐NCP‐ELISA based on the manufacturer's instructions (Euroimmun, product code EI 2606‐9601‐2 G).

In vitro neutralization assays toward authentic SARS‐CoV‐2 isolates were performed as previously described [40, 41]. The following viral isolates were utilized: Parental SARS‐CoV‐2 B.1 (FFM7/2020, GenBank ID: MT358643, [40]), SARS‐CoV‐2 XBB.1.5 (GISAID: EPI_ISL_19908355), SARS‐CoV‐2 JN.1 (GISAID: EPI_ISL_19621541), and SARS‐CoV‐2 KP3.1.1 (GenBank ID: PV653689). In brief, A549‐AT cells were incubated with serially diluted sera (1:2), which were preincubated with 4000 TCID_50_/mL of each SARS‐CoV‐2 variant. Forty‐eight hours after inoculation, the infected cells were observed for the formation of cytopathic effects (CPE) [42].

### 4.4 Statistical Analyses

Paired analyses were performed by nonparametric Wilcoxon signed‐rank testing (blood cell counts, T‐cell levels, IgG levels, and neutralization titers). Unpaired analyses of two groups were evaluated by parametric *t*‐tests (cytokine profiles) or nonparametric Mann–Whitney *U* testing (CTLA‐4). Multilevel comparisons of nonmatched subject data were made by one‐way ANOVA (parametric data; cytokine profiles) or Kruskal–Wallis testing (nonparametric; CTLA‐4 MFI) together with Tukey´s multiple comparison test. For matched subject data, Friedman testing together with Dunn´s multiple comparison test was applied (NT50 values). Reported adverse events among JN.1 vaccinated and JN.1 and influenza co‐vaccinated individuals were compared using Fisher's exact test. Fold‐increases were calculated by dividing the post‐values by the pre‐values. To avoid division by zero, the lower detection limit value of 0.03% for CD4 and 0.06% for CD8 T cells was added to the respective T‐cell levels before division. For correlation analyses, Spearman's correlation coefficients were determined. *p*‐values <0.05 were considered statistically significant. Multivariable linear regression analysis from log‐transformed values was performed to test the effects of age, sex, co‐administration of influenza vaccination, and a history of prior SARS‐CoV‐2 infection on vaccine‐induced immunity. GraphPad Prism 10.3.1 software was used to perform statistical analyses (GraphPad, San Diego, CA, USA).

## Author Contributions

C.D., R.U., T.S., and M.S. designed the study; C.D., R.U., D.B., S.C., and M.W. performed the experiments with support from S.B., J.E., and C.G.; C.G and F.H. contributed to patient recruitment and clinical data acquisition; C.D., R.U., and M.W analyzed the data; C.D. R.U., and M.S. visualized the data; C.D., R.U. M.W., and M.S. wrote the manuscript; all authors edited and approved the final manuscript.

## Ethics Approval for Human Studies and Consent Statement

The study was approved by the local ethics committee (“Ärztekammer des Saarlandes”, reference no. 76/20 including amendments). Written informed consent was given by all study participants.

## Conflicts of Interest

M.S. has received grant support from Astellas and Biotest to the organization Saarland University outside the submitted work, and honoraria for lectures from Biotest, Takeda, Qiagen, MSD, and served in advisory boards for Moderna, Biotest, MSD, and Takeda. M.W. has received grant support from Roche and Qiagen and honoraria for lectures from AstraZeneca and Qiagen. T.S. has received travel support from Biotest. The remaining authors declare no conflicts of interest.

## Supporting information




**Supporting File**: eji70232‐sup‐0001‐SuppMat.docx.

## Data Availability

The data underlying this article are available from the corresponding author upon reasonable request.
