# Supplementary material for: Induction of Humoral and Cellular Immunity After SARS‐CoV‐2 JN.1 Vaccination in Individuals With and Without Prior Infection
Source: Eur J Immunol. 2026 Jun 23;56(6):e70232. doi: 10.1002/eji.70232 (PMC13288449; doi:10.1002/eji.70232)
Supplement: Supplementary file 1 — Supporting File: eji70232‐sup‐0001‐SuppMat.docx. [file EJI-56-e70232-s001.docx]

# Supplement

# Induction of humoral and cellular immunity after SARS-CoV-2 JN.1 vaccination in individuals with and without prior infection

This supplement contains supplementary tables S1-S4, and one supplementary figure S1.

# Supplementary tables

## Table S1: Comparison of the quantitative changes in vaccine-induced humoral and cellular immunity towards the SARS-CoV-2 variants.

| **T cells** |  | **SARS-CoV-2 spike-specific** | | |  | p-value**^#^** |
| --- | --- | --- | --- | --- | --- | --- |
|  | **SEB** | **parental** | **XBB.1.5** | **JN.1** |  |  |
| Specific CD4 T cells (pre) | 3.9% (3.08%) | 0.05% (0.07%) | 0.06% (0.07%) | 0.05% (0.08%) |  | **p=0.016** |
| Specific CD4 T cells (post) | 4.15% (3.44%) | 0.14% (0.14%) | 0.13% (0.12%) | 0.14% (0.13%) |  | p=0.094 |
| Specific CD8 T cells (pre) | 6.94% (3.97%) | 0.05% (0.13%) | 0.04% (0.11%) | 0.04% (0.08%) |  | **p=0.019** |
| Specific CD8 T cells (post) | 7.39% (4.77%) | 0.14% (0.35%) | 0.14% (0.42 %) | 0.10% (0.48%) |  | p=0.693 |
| Fold change (IQR)^a^ | SEB | Parental spike | XBB.1.5 spike | JN.1 spike |  |  |
| Specific CD4 T cells | 1.02 (0.28) | 1.96 (0.90) | 1.97 (1.16) | 2.07 (1.37) |  | p=0.222 |
| Specific CD8 T cells | 1.04 (0.34) | 1.50 (1.33) | 1.51 (1.78) | 1.83 (2.07) |  | p=0.379 |
| **Antibody (Ab)** | **IgG** | **nAb parental** | **nAb XBB.1.5** | **nAb JN.1** | **nAb KP.3.1.1** |  |
| Spike-specific Ab (pre) | 1701 (1507) | 320 (520) | 20 (40) | 0 (20) | 10 (40) | **p<0.0001** |
| Spike-specific Ab (post) | 6530 (5334) | 800 (1600) | 100 (275) | 50 (140) | 100 (320) | **p<0.0001** |
| Fold change (IQR)^a^ |  |  |  |  |  |  |
| Spike-specific nAb | n. a. | 2 (1) | 4 (14) | 4 (10) | 4 (14) | **p<0.0001** |
| Spike-specific IgG | 2.26 (1.79) | n. a. | n. a. | n. a. | n. a. |  |

Values were calculated from samples before and after vaccination; ^a^the fold change was calculated as a ratio between post and pre-vaccination values; to avoid division by 0, the value 0.03% and 0.06% as the respective detection limits were added to each percentage of CD4 and CD8 T cells prior to division, respectively; ^#^p-value refers to comparison among spike-specific CD4 and CD8 T cells, and SARS-CoV-2-specific neutralizing activity, respectively; IQR, interquartile range; nAb, neutralizing antibodies.

## Table S2: Multivariable regression analyses of immune responses before SARS-CoV-2 JN.1 vaccination.

| **Dependent variables^a^** | **Age** | | **Sex** | | | **History of previous infection** | | |
| --- | --- | --- | --- | --- | --- | --- | --- | --- |
|  | Estimate (95% CI) | p-value | Estimate (95% CI) | | p-value | Estimate (95% CI) | | p-value |
|  |  |  | female [Ref] | male |  | no [Ref] | yes |  |
| IgG | 0.002 (-0.004 – 0.009) | 0.473 | 1 | 0.090 (-0.081 – 0.261) | 0.292 | 1 | **-0.231 (-0.421 – -0.042)** | **0.018** |
| NT50 parental | 0.016 (-0.028 – 0.059) | 0.471 | 1 | 0.996 (-0.137 – 2.128) | 0.083 | 1 | -0.363 (-1.618 – 0.892) | 0.560 |
| NT50 XBB.1.5 | 0.025 (-0.026 – 0.076) | 0.322 | 1 | 0.706 (-0.615 – 2.026) | 0.285 | **1** | **-1.505 (-2.968 – -0.042)** | **0.044** |
| NT50 JN.1 | 0.018 (-0.013 – 0.050) | 0.244 | 1 | 0.609 (-0.205 – 1.423) | 0.137 | **1** | **-1.713 (-2.615** **– -0.811)** | **<0.001** |
| NT50 KP.3.1.1 | 0.010 (-0.031 – 0.052) | 0.616 | 1 | 0.892 (-0.178 – 1.962) | 0.099 | **1** | **-2.383 (-3.569 – -1.198)** | **<0.001** |
| CD4 T cells parental | 0.001 (-0.008 – 0.009) | 0.899 | 1 | 0.025 (-0.198 – 0.247) | 0.823 | 1 | 0.134 (-0.129 – 0.397) | 0.308 |
| CD4 T cells XBB.1.5 | 0.002 (-0.009 – 0.012) | 0.744 | 1 | 0.013 (-0.251 – 0.278) | 0.919 | 1 | -0.057 (-0.370 – 0.256) | 0.714 |
| CD4 T cells JN1 | -0.003 (-0.014 – 0.009) | 0.664 | 1 | 0.064 (-0.235 – 0.363) | 0.665 | 1 | -0.261 (-0.612 – 0.091) | 0.141 |
| CD8 T cells parental | 0.002 (-0.015 – 0.019) | 0.834 | 1 | 0.128 (-0.294 – 0.551) | 0.541 | 1 | 0.312 (-0.188 – 0.812) | 0.212 |
| CD8 T cells XBB | -0.004 (-0.020 – 0.013) | 0.639 | 1 | 0.060 (-0.349 – 0.468) | 0.768 | 1 | 0.419 (-0.065 – 0.902) | 0.087 |
| CD8 T cells JN.1 | -0.002 (-0.021 – 0.016) | 0.784 | 1 | 0.077 (-0.382 – 0.536) | 0.735 | 1 | 0.214 (-0.325 – 0.752) | 0.425 |

Shown are p-values of multivariable linear regression analyses with log(10) transformed values; ^a^parameters refer to spike-specific IgG [BAU/ml], neutralizing activity (NT50), and spike-specific CD4 and CD8 T cells [%], females and no prior infection as references for categorical parameters; NT50 parental refers to the FFM7 strain. 95% CI, 95% confidence interval.

## Table S3: Adverse events depending on co-administration of the influenza vaccine

| Adverse events | | all | JN.1/influenza | JN.1 only | | p-value^a^ | |
| --- | --- | --- | --- | --- | --- | --- | --- |
|  |  | n=37 | n=25 | n=12 | |  |  |
| General adverse events, n (%) | | 26 (70.3) | 14 (56.0) | 12 (100.0) | | **p=0.007** | |
|  | no adverse events | 11 (29.7) | 11 (44.0) | 0 (0) | | **p=0.006**^b^ | |
|  | local only | 8 (21.6) | 2 (8.0) | 6 (50.0) | |  | |
|  | systemic only | 4 (10.8) | 2 (8.0) | 2 (16.7) | |  | |
|  | local and systemic | 14 (37.8) | 10 (40.0) | 4 (33.0) | |  | |
| Antipyretic medication, n (%) | | 5 (13.5) | 5 (20.0) | 0 (0) | | p=0.152 | |
| Local adverse event, n (%) | | 22 (59.5) | 12 (48.0) | 10 (83.3) | | p=0.073 | |
|  | pain at injection site | 21 (56.8) | 12 (48.0) | 9 (75.0) | | p=0.166 | |
|  | swelling the injection site | 6 (16.2) | 3 (12.0) | 3 (12.0) | | p=0.367 | |
| Systemic adverse events, n (%) | | 14 (37.8) | 12 (48.0) | 6 (24.0) | | p>0.999 | |
|  | fever | 0 (0) | 0 (0) | | 0 (0) | p>0.999 |  |
|  | fatigue | 13 (35.1) | 9 (36.0) | | 4 (33.0) | p>0.999 |  |
|  | headache | 10 (27.0) | 8 (32.0) | | 2 (16.7) | p=0.445 |  |
|  | chills | 2 (5.4) | 1 (4.0) | | 1 (8.3) | p>0.999 |  |
|  | GI effects | 3 (8.1) | 2 (8.0) | | 1 (8.3) | p>0.999 |  |
|  | myalgia | 5 (13.5) | 4 (16.0) | | 1 (8.3) | p>0.999 |  |
|  | arthralgia | 6 (16.2) | 5 (20.0) | | 1 (8.3) | p=0.641 |  |

^a^refers to difference between JN.1/influenza co-administration and JN.1 only using Fisher’s exact test or ^b^X^2^-test; response rate of the questionnaire was 100%.

## Table S4: Antibodies used for flow-cytometric analyses

| **Target** | **Conjugate** | **Clone** | **Isotype** | **Host Organism** | **Dilution** | **RRID** |
| --- | --- | --- | --- | --- | --- | --- |
| CD4 | APC-H7 | SK3 | IgG1k | mouse | 1:33.3 | AB_1645732 |
| CD8 | PerCP | SK1 | IgG1 k | mouse | 1:12.5 | AB_2868802 |
| CD69 | PE-Cy7 | L78 | IgG1 k | mouse | 1:33.3 | AB_1937286 |
| CTLA-4 | APC | BNI3 | IgG2a k | mouse | 1:50 | AB_398615 |
| IFNγ | FITC | 4S.B3 | IgG1 k | mouse | 1:100 | AB_395473 |
| IL-2 | PE | MQ1-17H12 | IgG2a k | rat | 1:50 | AB_397231 |
| TNF | V450 | MAb11 | IgG1 k | mouse | 1:20 | AB_10646031 |

All antibodies were purchased from BD, Heidelberg, Germany; dilution refers to antibody concentration in the staining reaction; RRID, research resource identifier.

# Supplementary figure

## Figure S1


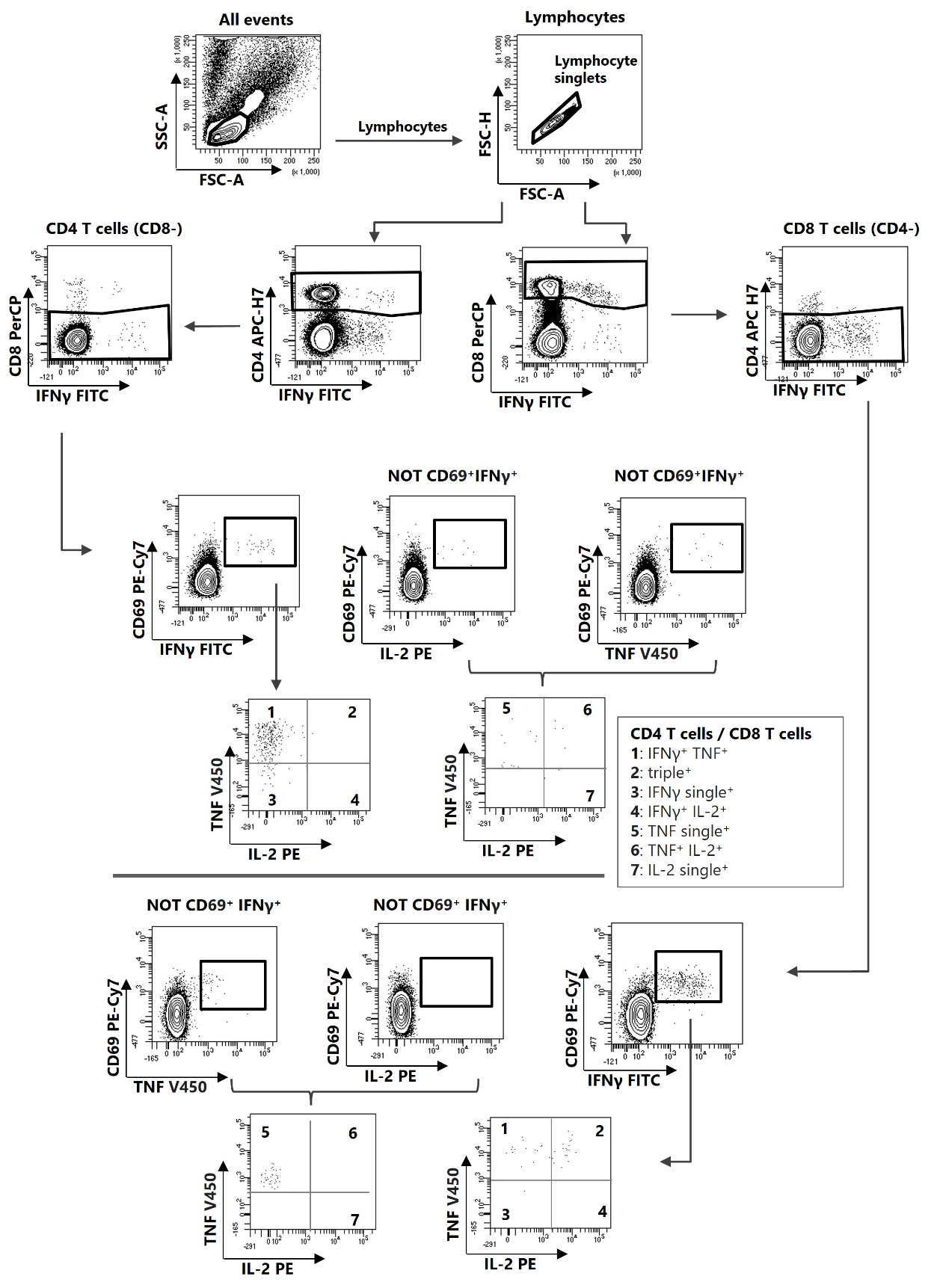


**Figure S1: Gating strategy for the identification and characterization of spike-specific T cells.** All measured events were displayed by FSC (forward scatter) and SSC (side scatter), after backgating on CD4, to identify the lymphocyte population. Exclusion of doublets was performed by display of FSC height and area, followed by removal of events with unspecific fluorescence in channel V500. CD4 and CD8 populations were then displayed for CD69 and IFNγ expression to identify CD69^+^IFNγ^+^ events, indicated by square gates, and for calculation of percentages, respectively. These cells were evaluated for expression of cytotoxic T-lymphocyte protein 4 (CTLA-4). Boolean Gating was applied on CD69^+^IFNγ^+^ cells and CD69^+^IFNγ^-^ but IL-2^+^ or TNF^+^ cells to analyse concurrent cytokine expression.
